# Age-dependent pathogenic characteristics of SARS-CoV-2 infection in ferrets

**DOI:** 10.21203/rs.3.rs-131380/v2

**Published:** 2021-03-29

**Authors:** Young-Il Kim, Kwang-Min Yu, June-Young Koh, Eun-Ha Kim, Se-Mi Kim, Eun Ji Kim, Mark Anthony Casel, Rare Rollon, Seung-Gyu Jang, Min-Suk Song, Su-Jin Park, Hye Won Jeong, Eung-Gook Kim, Ok-Jun Lee, Younho Choi, Shin-Ae Lee, Su-Hyung Park, Jae U. Jung, Young Ki Choi

**Affiliations:** College of Medicine and Medical Research Institute, Chungbuk National University; Chungbuk National University; Graduate School of Medical Science and Engineering, Korea Advanced Institute of Science and Technology (KAIST); Chungbuk National University; Chungbuk National University; College of Medicine and Medical Research Institute, Chungbuk National University; College of Medicine and Medical Research Institute, Chungbuk National University; College of Medicine and Medical Research Institute, Chungbuk National University; College of Medicine and Medical Research Institute, Chungbuk National University; Chungbuk National University; Division of Applied Life Science and Research Institute of Life Sciences, Gyeongsang National University; Department of Internal Medicine, Chungbuk National University College of Medicine; Chungbuk National University; Chungbuk National University; Lerner Research Institute, Cleveland Clinic; Lerner Research Institute, Cleveland Clinic; Korea Advanced Institute of Science and Technology; Lerner Research Institute, Cleveland Clinic; Chungbuk National University

**Keywords:** COVID-19, age-dependent, ferrets, pathogenesis, clinical manifestations

## Abstract

While the seroprevalence of SARS-CoV-2 in healthy people does not differ significantly among age groups, those aged 65 years or older exhibit strikingly higher COVID-19 mortality compared to younger individuals. To further understand differing COVID-19 manifestations in patients of different ages, three age groups of ferrets were infected with SARS-CoV-2. Although SARS-CoV-2 was isolated from all ferrets regardless of age, aged ferrets (≥ 3 years old) showed higher viral loads, longer nasal virus shedding, and more severe lung inflammatory cell infiltration and clinical symptoms compared to juvenile (≤ 6 months) and young adult (1–2 years) groups. Transcriptome analysis of aged ferret lungs revealed strong enrichment of gene sets related to type I interferon, activated T cells, and M1 macrophage responses, mimicking the gene expression profile of severe COVID-19 patients. Thus, SARS-CoV-2-infected aged ferrets highly recapitulate COVID-19 patients with severe symptoms and are useful for understanding age-associated infection, transmission, and pathogenesis of SARS-CoV-2.

## Introduction

Coronavirus Disease 2019 (COVID-19) is caused by a novel emerging Severe Acute Respiratory Syndrome Coronavirus 2 (SARS-CoV-2)^1^. Since the first outbreak in China in November 2019, COVID-19 has spread rapidly and globally with high mortality, resulting in a serious threat worldwide. Thus, the World Health Organization (WHO) declared SARS-CoV-2 a pandemic on March 11, 2020^2^. SARS-CoV-2 is the third identified epidemic coronavirus transmitted from animals to humans since 2002, with the others being SARS-CoV^3^ and Middle East Respiratory Syndrome Coronavirus (MERS-CoV)^4^. However, the magnitude of impact of SARS-CoV-2 infection is by far the greatest due to the significantly larger number of human cases, and consequently, higher mortality.

Despite strict precautionary measures, such as social distancing policies and restriction of social gatherings, the number of SARS-CoV-2 infections continues to grow exponentially with a proportional increase in mortality^5^. Fortunately, several licensed COVID-19 vaccines with high efficacy have been developed in record time. It is expected that by the end of next year all populations will have access to these safe and effective vaccines. However, given the strong correlation between severe COVID-19 and increasing age, it is imperative to understand the etiology of severe disease and determine the most effective treatment strategies for high-risk groups. Although patients with COVID-19 are distributed among all age groups, the majority of patients with severe COVID-19 fall within the 30 to 79 years of age (87%) grouping, while younger patients aged 10 to 19 years only comprised about 1% of total cases^6^. Moreover, higher morbidity and mortality rates have consistently been observed in aged human populations throughout the COVID-19 pandemic^7^. Similarly, mouse-adapted SARS-CoV showed strong age-dependent disease phenotypes in a mouse model^8,9^. Furthermore, when compared to the young adult population, the aged population is generally more susceptible to respiratory infections and shows a poor prognosis^10^. Thus, this indicates that age is a critical factor for COVID-19 viral disease severity.

In addition to the various clinical symptoms reported for COVID-19, systematic experimental studies are needed to further dissect the diverse disease manifestations among different age groups. While human clinical studies are highly valuable, a number of limitations, including ethical issues, behavioral and environmental variables, and the medical history of the patients, may impede identification of the fundamental cause of the disease in a timely manner. Hence, this necessitates the development of an appropriate animal model to aid in understanding transmission and pathogenesis, as well as elucidating host immune responses to SARS-CoV-2. The current hACE2 transgenic mouse model, which expresses the human ACE2 entry receptor for SARS-CoV-2, showed weight loss and virus replication in lungs following SARS-CoV-2 infection. Although some mice showed neurological symptoms which lead to fatal infection in a virus dose-dependent manner^11,12^, other clinical symptoms of infection, such as body temperature, sneezing, coughing, and lethargy, are difficult to monitor in mouse model. Further, the neurologic-related mortality also brings the suitability of this animal model into debate, as central nervous system infection is rarely observed in COVID-19 patients, underscoring the need for an animal model that could better represent the human disease state. Following the fortuitous discovery that ferrets have natural susceptibility to human influenza viruses, ferrets have been recognized as a useful animal model for study of respiratory viruses, such as respiratory syncytial virus, parainfluenza viruses, and SARS coronavirus^13-16^. Ferrets have a respiratory tract histo-anatomically analogous to that of humans, with similar anatomic proportions of the upper and lower respiratory tracts, density of submucosal glands in the bronchial wall, and number of generations of terminal bronchioles^15^, further supporting the adequacy of this model for study of human respiratory viral infections.

In order to address numerous critical scientific questions ranging from basic virology to the development and assessment of novel drugs and vaccines for COVID-19, we have recently established a ferret model for SARS-CoV-2 infection and transmission that highly recapitulates pathological aspects of the human infection^17^. SARS-CoV-2-infected ferrets exhibited elevated body temperatures and virus replication was readily detected; infected ferrets shed the virus through nasal washes and in saliva, urine, and fecal specimens; SARS-CoV-2 was readily transmitted to naïve direct-contact ferrets, but less efficiently to naïve indirect-contact ferrets; and acute bronchiolitis was observed in infected lungs^17^. Thus, SARS-CoV-2 replicates efficiently in the respiratory tracts of ferrets without prior adaption. Compared to small mammalian models, various clinical signs found in COVID-19 patients are also observable in ferrets, including elevated body temperatures, nasal discharge, sneezing, and shedding of the virus through nasal washes, saliva, urine, and feces^17^. Moreover, investigation of viral transmission of SARS-CoV-2 revealed the ability to spread to naïve ferrets through direct-contact and indirectly through respiratory droplets^17^. Also, we have recently developed an aged ferret infection model (≥4 years old, equivalent to 70 years old in humans) for the emerging human severe fever with thrombocytopenia syndrome virus (SFTSV) that fully recapitulates human clinical manifestation^18^. SFTSV infection exhibits a severe clinical manifestation and increased fatality rate in patients 50 years and older, which was recapitulated in SFTSV-infected aged ferrets, as evidenced by severe symptoms, such as high temperature, weight loss, severe thrombocytopenia, and death.

To further explore the age-related disease severity observed in COVID-19 patients, we infected ferrets divided into three different age groups with SARS-CoV-2: ferrets under 6 months to simulate juveniles/children (G1), 1 to 2-year-old ferrets to simulate young adults (G2), and ferrets more than 3 years old to simulate patients over 50 years old (G3). To compare disease severity among these three groups, clinical symptoms, viral load in the respiratory tract, and lung histopathology were examined. Furthermore, RNA sequencing (RNA-seq) analysis was performed with lung tissues from SARS-CoV-2-infected young adult or aged ferrets to compare differences in global and dynamic gene expression. As a result, the aged (G3) ferret group showed higher viral load and more severe clinical symptoms than juvenile (G1) and young adult (G2) ferret groups, and expressed high levels of gene sets related to type I interferon (IFN), activated T cells, and M1 macrophage responses. Thus, SARS-CoV-2-infected aged ferrets considerably resemble COVID-19 aged patients with severe symptoms. This demonstrates the ability of this animal model to recapitulate the age-dependent etiology of severe COVID-19, and indicates the feasibility of its use in the development of novel therapeutics and vaccines for the exact target group.

## Results

### Clinical features of SARS-CoV-2 infection among ferrets of different ages

**Figure 1:**
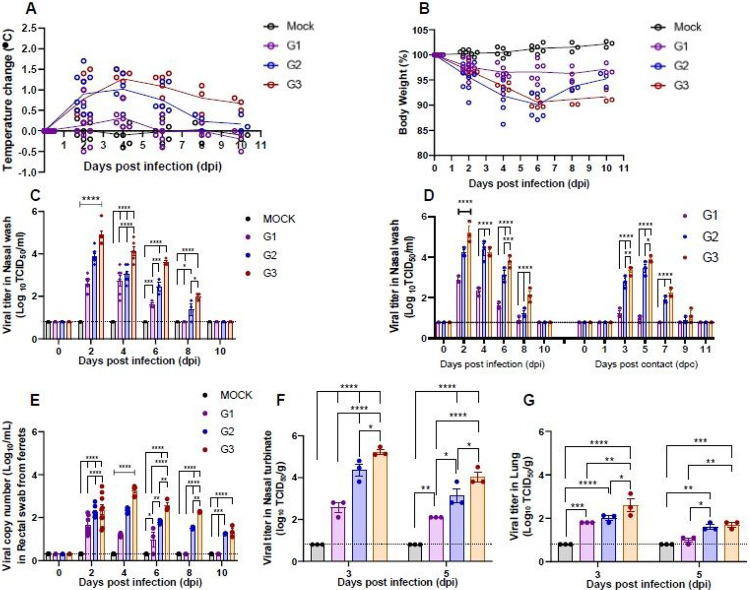
Pathogenicity of SARS-CoV-2 among different ages of ferrets. Groups of ferrets [under 6 months (G1), 1 to 2 years old (G2), and more than 3 years old (G3); n=9 per group] were inoculated with 105.8 TCID50 of NMC-nCoV02 strain by the intranasal route. All groups were observed for morbidity and mortality for 10 days. The temperature change (A) and weight loss (B) are shown. To compare virus growth in respiratory and gastrointestinal tracts, nasal washes (C-D) and rectal swabs (E) were collected at 0, 2, 4, 6, 8, and 10 dpi. Transmission properties of SARS-CoV-2 in ferrets of different ages were compared by co-housing naïve ferrets (n=9/group) in direct contact (DC)(n=3/group) with each group of infected ferrets (n=3/group) starting two days after the primary infection, followed by measurement of their viral titers from nasal washes on day 1, 3, 5, 7, 9, and 11 post contact (D). Nasal turbinate (F) and lung (G) tissues were collected to recover infectious virus from infected ferrets (n=3) at 3 and 5 dpi. Infectious viral titers in nasal washes and tissue specimens (C, D, F, and G) were measured in Vero Cells, and viral RNA copy numbers in rectal swabs were quantitated using real-time PCR (E). Asterisks indicate statistical significance compared with each sample by two-way ANOVA Tukey's multiple comparisons test (* indicates p < 0.05, ** indicates p < 0.01, *** indicates p < 0.001 and **** indicates p < 0.0001).

In order to elucidate the clinical manifestations of SARS-CoV-2 infection across age groups, ferrets (n=9/group) were inoculated with 10^5.8^ of 50% tissue culture infective dose (TCID_50_)/mL of NMC-nCoV02 strain through the intranasal (IN) route. While ferrets less than 6 months of age (G1) showed no increase in temperature, both the G2 (1-2 years old) and G3 (more than 3 years old) groups of SARS-CoV-2 infected ferrets showed elevated temperatures at 2-6 dpi, where the G3 group showed a prolonged elevated temperature even at 10 dpi (Fig. 1A). This trend was also associated with changes in body weight, where the G1 group showed less than 5% weight loss during the entire SARS-CoV-2 infection period, while the G2 and G3 groups showed a maximal 10% weight loss at 6 dpi, followed by a rapid recovery of the G2 group from 6 dpi but not the G3 group (Fig. 1B). To compare clinical manifestations of SARS-CoV-2 infection, we developed an arbitrary scoring method to describe clinical symptom (CS) values based on a 20-minute observation period of cough, rhinorrhea, and reduced activity. These CS values were compared among ferret groups as described in Table 1 and Table S1. The G2 and G3 groups showed the highest CS values of 4.17 and 4.67 at 4 dpi, respectively, while the G1 group showed a maximal CS value of 1 at 2 dpi before quickly recovering within 4 days, thus exhibiting a mild to asymptomatic infection (Table 1 and Table S1). Particularly, the G3 group showed a prolonged period of high CS value that lasted until 10 dpi. In addition, aged contact ferrets showed the highest and most prolonged CS value compared to young adult contact ferrets. However, juvenile contact ferrets did not show any symptoms of clinical disease during the course of infection (Table S2). Of note, there was no loss in body weight among contact groups. While a light elevation of body temperatures was observed among contact ferrets co-housed with aged and young adult groups, no increase in body temperatures was noted in contact ferrets in the juvenile group (Fig. S1). Collectively, these results revealed that aged ferrets exhibit more severe and persistent clinical features of SARS-CoV-2 infection compared to younger ferrets.

### Comparison of viral titer and shedding period among different age groups of ferrets

To evaluate whether the clinical features seen in the ferret groups were associated with the degree of virus replication, we measured infectious viral titers from nasal washes (Fig. 1C). SARS-CoV-2 was isolated from all infected ferrets, regardless of their ages, from 2 to 6 dpi, whereas ferrets in the G2 and G3 groups continued to shed infectious virus until 8 dpi. Comparison of viral titers revealed that the G3 group showed significantly higher viral titers (3.5 to 4.9 log_10_TCID_50_/mL) from 2 to 6 dpi than the G1 and G2 groups. While the G2 group showed higher viral titers at 2 dpi than the G1 group, comparable viral titers were observed thereafter. These results clearly demonstrate that aged ferrets (G3) shed higher amounts of infectious virus in nasal discharges for a longer period of time than younger ferrets.

Because gastrointestinal involvement has also been documented during coronavirus infections of animals and humans^19,20^, we collected fecal specimens from the infected ferrets and performed qRT-PCR to assess SARS-CoV-2 viral loads and shedding periods from the intestines of ferrets of different ages (Fig. 1E). While viral RNAs were detected in fecal specimens of all three groups from 2 to 4 dpi, the G3 group showed the highest viral RNA copy number from 2 to 8 dpi, followed by the G2 group. On the other hand, the G1 group showed a small peak of viral RNA at 2 dpi, which rapidly declined thereafter to an undetectable range at 6 dpi. To further evaluate the viral titer in respiratory organs of infected animals, three ferrets from each group were euthanized at both 3 and 5 dpi for measurement of viral titers in the nasal turbinate and lungs. As expected, the G3 group showed the highest viral titers among the groups at a maximum of 5.2 log_10_TCID_50_/g in nasal turbinate at 3 dpi, and the G2 group also showed significantly high viral titers compared with the G1 group (Fig. 1F). Consistently, the G3 group showed a significantly higher viral titer (2.6 log_10_TCID_50_/g) in lung tissues compared with the G1 and G2 groups at 3 dpi (Fig. 1G). These results demonstrate that the aged ferret group shows significantly higher SARS-CoV-2 titers in the respiratory tract compared to juvenile and young adult ferrets, correlating with the clinical disease severity.

### Comparison of SARS-CoV-2 transmission in ferrets by age

As viral titers differed significantly among different age groups, naïve ferrets (n=3/group) were co-housed and placed in direct contact (DC) with infected ferrets two days after the primary infection to compare the transmission efficiency of SARS-CoV-2 (Fig. 1D, Fig. S1 and Table S2). Virus was detectable in nasal washes of the DC ferrets as early as 3 days post-contact (dpc), and ferrets co-housed with the G3 group showed the highest viral titers at all time points with a peak of 4.10 log_10_ TCID_50_/mL at 5 dpc. The DC ferrets co-housed with G2 and G3 groups shed infectious virus up to day 9 post-contact, whereas the DC ferrets exposed to G1 ferrets showed the lowest viral titers all throughout the co-housing period, reaching an undetectable range of infectious virus after 7 dpc (Fig. 1C, right panel). This showed that as the aged ferret group harbored high SARS-CoV-2 titers in their respiratory tracts, they were able to effectively transmit the virus to naïve ferrets by direct contact. As seen with children who appear to be less infectious than adults infected with SARS-CoV-2 due to their mild clinical manifestation of disease^21^, the infected juvenile ferret group carried low virus titer and was not readily infectious to naïve ferrets upon direct contact.

### Differential lung histopathology of infected ferrets of different ages

**Figure 2:**
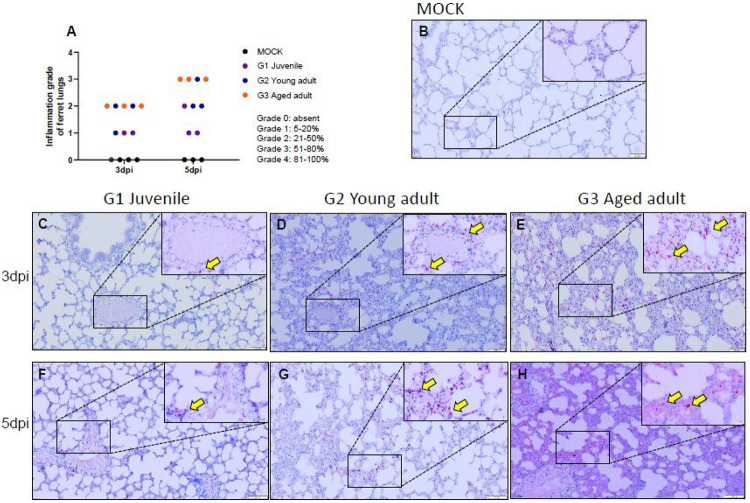
RNAscope in situ hybridization in lung tissues of SARS-CoV-2 infected ferrets. To detect the SARS-CoV-2 RNA (Spike gene) in lung tissues, RNA scope in situ hybridization was performed using a Spike-specific probe (Advanced Cell Diagnostics, cat # 848561) and visualized using RNAscope 2.5 HD Reagent Kit RED (Advanced Cell Diagnostics, cat # 322360). The degree of inflammation in lung tissue samples was compared (%) (A). SARS-CoV-2 spike RNA-positive cells (Yellow arrows) in lung tissues of mock infected (B), juvenile ferrets (≤ 6 months, G1 group) (C, F), young adult (1 ≤ age ≤ 2 years, G2 group) (D, G), and aged ferrets (3-year ≤ ages) (E,H). Lung sections comparing different age groups at 3 dpi (C, D, and E) and at 5 dpi (F, G, and H).

COVID-19 has most commonly been shown to be associated with a spectrum of lung damage. The aged ferrets showed considerable inflammation affecting most parts of lungs compared to the juvenile and young adult ferrets that showed only mild to moderate inflammation. Notably, all aged ferrets showed more than 50% lung damage at 5 dpi, (Fig. 2A and Fig S3). To further compare the extent of pulmonary damage among ferrets of different ages, RNAscope *in situ* hybridization and histopathological examination were conducted (Fig. 2, Fig. S2, and S3). Based on RNAscope analysis of SARS-CoV-2, we could find more virus infected cells in young adult and aged ferrets compared with the juvenile group (Fig. 2C-H). However, there was no significant difference between the young adult and aged ferrets at 5 dpi (Fig. 2G and H) although the aged ferrets showed higher numbers of viral RNA-positive cells compared with the young adult group at 3 dpi (Fig. 2D and E). Further, this was reflected by virus titers in the lungs (Fig. 1G). The G2 group (Fig. S3 E-F) displayed only moderate pathological changes in the lung in comparison to the G1 group (Fig. S3 A-B). In contrast, analysis of lungs from the G3 group revealed increased severity of pathological features highlighted by increased inflammatory cell infiltration and alveolar septa being widened, edematous, and congested (Fig. S3 I-L). To rule out differential ACE2 expression among age groups, which may affect disease manifestation and virus replication kinetics in ferrets, ACE2 RNAscope analysis was performed on ferret lung sections. This showed that there was no detectable difference of the ACE2 expression among different age groups (Fig. S4A-H). These results demonstrate that the severity of lung damage is closely associated with the number of SARS-CoV-2 RNA-positive cells in the lungs, but not with ACE2 receptor expression levels.

### Differential SARS-CoV-2 antibody neutralization titers and serum IgG antibody among ferrets of different ages

**Figure 3:**
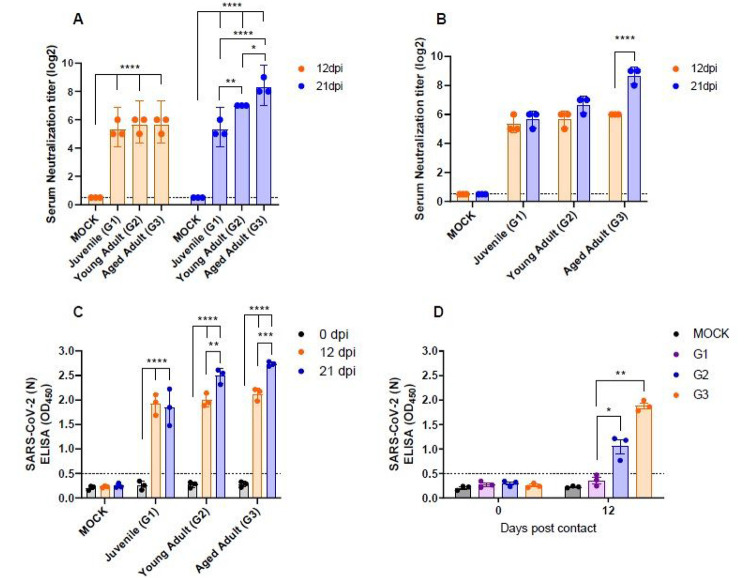
Serum neutralizing antibody (NAb) titers and IgG ELISA in ferrets. The NAb titers against SARS-CoV-2 NMC-nCoV02 (100 TCID50) among different age groups was measured in Vero cells with serially diluted ferret sera collected at 12 and 21 dpi (A). Variation of NAb titers between groups at 12 and 21 dpi (B). The IgG titers among different age groups in serially diluted sera collected at 12 and 21 dpi (C), IgG titers in contact groups at 12 dpi (D). Each test was repeated three times and data are presented as geometric mean ± SEM. Asterisks indicate statistical significance compared with primary infection sera as evaluated by two way ANOVA Tukey’s multiple comparisons tests (* indicates p < 0.05, ** indicates p < 0.01, and *** indicates p < 0.0001) (A, C and D), and two way ANOVA Sidak’s multiple comparisons tests (* indicates p < 0.05, ** indicates p < 0.01, and *** indicates p < 0.0001) (B).

To compare the serum neutralization antibody (NAb) titers among ferrets of different ages, blood was collected from each group of ferrets at 12 and 21 dpi. At 12 dpi, all tested ferrets showed NAb titers of 32-64 without significant differences among the groups. However, at 21 days, the NAb titers were at least four-fold higher in the G3 group (GMT 256) than in the G1 group (GMT 64) (Fig. 3A). Furthermore, the G3 group showed significantly increased NAb titers at 21 dpi compared to 12 dpi, suggesting continuous activation of the immune response as long as 21 dpi (Fig. 3B). To further evaluate the serum IgG antibody titer, we conducted an ELISA on serum from each ferret. ELISA results revealed that the young adult (G2) and aged adult (G3) groups showed increased anti-SARS-CoV-2 antibody titers at 21 dpi (Fig. 3C). However, there were no statistical differences of anti-SARS-CoV-2 antibody titers in the juvenile group at 12 and 21 dpi. Two ferrets of the juvenile group (G1, n=3) exhibited a decrease of anti-SARS-CoV-2 antibody titers, and only one ferret showed a similar anti-SARS-CoV-2 antibody titer at 12 dpi. In addition, IgG antibody titers in serum of direct contact ferret groups of different ages were measured by ELISA. While the juvenile contact group showed only minimally detectable IgG at 12 dpi (Fig. 3D), young adult or aged ferrets demonstrated moderate or marked increase in IgG titers, respectively. This indicates that similar to severe COVID-19 patients^22^, aged ferrets display severe clinical symptoms and high viral titers in the respiratory tract as well as high serum NAb and IgG responses.

### Transcriptional profile of immune-related genes in lung tissues of SARS-CoV-2-infected ferrets

**Figure 4:**
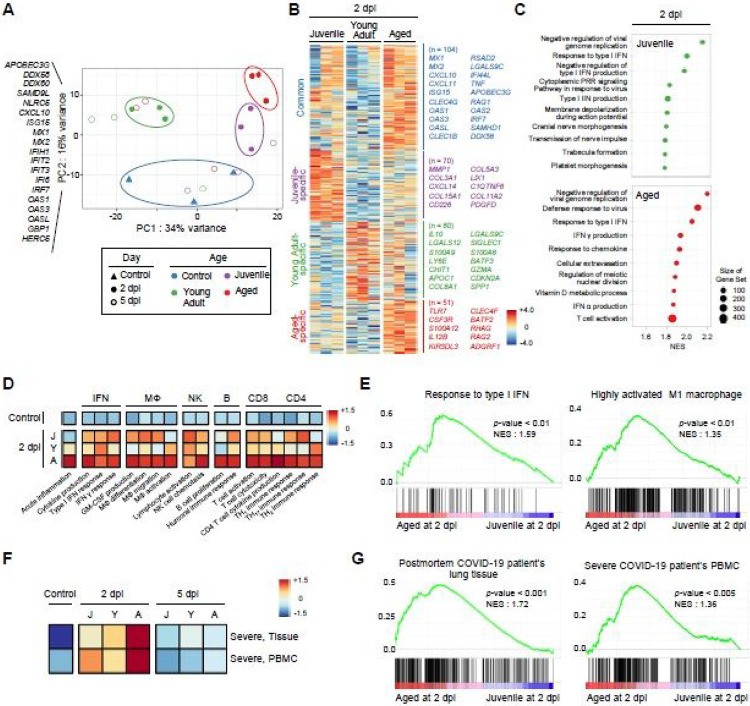
Transcriptional profile of immune-related genes in lung tissues of SARS-CoV-2-infected ferrets. Principal component analysis (PCA) scatter plot of gene expression of the SARS-CoV-2 infected ferrets showing distinct cluster according to ages, associated with interferon stimulated signature (A). Heatmap of age-specific differentially expressed genes (DEGs) compared to control ferrets shows that genes related to innate immune response r were upregulated in 2 dpi (B). The ‘Common’ gene sets were composed of genes differentially upregulated in more than two groups, while ‘Juvenile-specific’, ‘Young adult-specific’ and ‘Aged-specific’ gene sets were composed of genes uniquely upregulated in each group. Representative immune-related genes were listed next to the heatmap. Bar plots with normalized enrichment score (NES) from enrichment analysis of representative Gene Ontology (GO) biological pathway shows anti-viral immune responses were enrich in both 2 dpi (C). Heatmap of gene set variation analysis (GSVA) with immune related GO biological pathway shows that various immune responses were upregulate in aged compared to juvenile or young adult ferrets (D). J, Juvenile; Y, Young adult; A, Aged. Gene set enrichment analysis (GSEA) of gene sets related to type I IFN response and highly activated M1 macrophage between juvenile (G1) and aged (G3) ferrets at 2 dpi (E). GSVA and GSEA with gene sets from severe COVID-19 patients between young (G1) and aged (G3) ferret at 2 dpi (F-G).

To gain a comprehensive understanding of the transcriptional profile among ferrets of different ages following SARS-CoV-2 infection, we performed RNA sequencing (RNA-seq) analysis of lung tissues from G1, G2, and G3 ferret groups at 2 and 5 dpi, and compared the results with those of non-infected middle-aged ferrets (control, n=3). We first analyzed the overall variation of the samples using principal component analysis (PCA). Distinct clusters were observed among G1, G2, and G3 groups at 2 dpi (filled circle, lll), which were also clearly separated from that of control ferrets (filled triangle, p) (Fig. 4A). Intriguingly, age-dependent clustering at 2 dpi was largely attributed to principal component (PC) 2, which, in the majority, was composed of interferon-stimulated genes (ISGs), such as IRF7, ISG15, and OAS1 (Fig 4B, Table S3). These findings indicate that genes related to IFN responses are highly enriched in aged ferrets compared to juvenile ferrets during the early stage of SARS-CoV-2 infection (2 dpi). In contrast, samples at 5 dpi (open circle) did not display any particular clustering by age in PCA. This phenomenon could be explained by the recovery of several animals (one ferret in each of the G2 and G3 groups, and two ferrets in the G1 group) from SARS-CoV-2 infection, which were clustered with the control group.

To gain deeper insight into the divergent immune responses of SARS-CoV-2-infected ferrets with age, we identified differentially expressed genes (DEGs) in each group (Table S4). Compared to non-infected control ferrets, a total of 104 genes, including a number of ISGs (*IFI44L, ISG15, MX1, MX2, OAS1, OAS2,* OAS3, and *OASL*), genes related to chemokines (*CXCL10* and *CXCL11*), and interferon regulatory transcription factor (*IRF7*), were commonly upregulated in SARS-CoV-2 infected-ferrets at 2 dpi regardless of age (Fig. 4B). On the other hand, 70, 80, and 51 genes were uniquely upregulated in G1, G2, and G3 groups at 2 dpi, respectively. Notably, the DEGs that were specifically upregulated in the G3 group at 2 dpi included genes related to activation of the innate immune response (*CLEC4F, CSF3R, S100A12,* and *TLR7*) and initiation of the adaptive immune response (IL12B), whereas genes associated with tissue remodeling (*COL3A1, COL5A3, COL11A2, and MMP1*) were highly enriched in the G1 group at 2 dpi (Fig. 4B). At 5 dpi, various ISGs (*IFI6, IFI44,* and *IFI44L*) and innate immunity-associated genes (CLEC4G, RSAD2, and TRIM22) were commonly upregulated in all SARS-CoV-2 infected ferrets (Fig. S5A). In particular, genes related to activated T cell responses, including *CD69, IL7R* and *PDCD1*, were markedly enriched in the G3 group compared to the G1 and G2 groups.

Gene Set Enrichment Analysis (GSEA) revealed that DEGs of the G3 aged group were highly enriched with gene sets related to the anti-viral innate immune response as well as the adaptive immune response (T cell activation) at 2 dpi (Fig. 4C). These immune activation features were maintained even at 5 dpi (Fig. S5B). In contrast, tissue remodeling-related gene sets, such as trabecula formation, chondrocyte proliferation and cornification, were highly enriched in the G1 juvenile group both at 2 and 5 dpi (Fig. 4C and Fig. S5B). These gene sets may be closely associated with matrix remodeling during the recovery of lung epithelial injury in the juvenile group.

Furthermore, Gene Set Variation Analysis (GSVA) with public gene sets revealed that genes related to B cell response and T cell response were predominantly enriched in the aged group (G3) at 2 dpi, compared to the juvenile (G1) or the young adult (G2) ferrets (Fig. 4D), which is in agreement with a recent clinical study that showed stronger antibody and T cell responses in severe COVID-19 patients than in patients with mild disease^23,24^. Moreover, other immune-related gene sets, including IFN response, macrophage activation and NK cell activation, were also highly enriched in the aged group at 2 dpi (Fig. 4D). Notably, gene sets related to type I IFN responses and activated M1 macrophages were significantly enriched in aged ferrets at the earlier stage of SARS-CoV-2 infection (Fig. 4E), suggesting a marked activation of immune response-associated inflammation promptly followed by the initial infection in aged ferrets. Moreover, chemokines, such as CCL4, CCL5 and CXCL10, were markedly upregulated in most aged ferret at 2 dpi compared to other groups. Similarly, high expressions of IFNB1, IFNG, IL1B, IL2, IL7, and TNF were also observed at 2 dpi (Fig. S6). At 5 dpi, chemokine and cytokine expressions then returned to normal levels. These data suggest that aged ferrets have increased expression of inflammatory cytokines and chemokines during the early phase of infection. Finally, we investigated whether the SARS-CoV-2-infected aged ferrets also reproduced the natural course of severe COVID-19 as seen in humans. In fact, the gene sets upregulated in postmortem lung tissue of COVID-19 patients^25^ or PBMCs from severe COVID-19 patients^26^ were also highly enriched in aged ferrets (G3), especially at 2 dpi, compared to the juvenile (G1) and young adult (G2) ferrets (Fig. 4F and G). These transcriptional profiles of immune-related genes indicate that the SARS-CoV-2-infected ferret model reflects the immunological properties of age-dependent severe COVID-19 in humans.

## Discussion

SARS-CoV-2 has already had devastating effects on the global community affecting myriad aspects of our lives. While effective vaccines will be readily available soon, the exact timeline is unclear and although there is finally hope on the horizon, things are expected to get worse in the meantime, as thousands of people die every day and are separated from their families, pushing the national health care system to the brink. Further, emergence of new variants, such as Brazil variant (P.1), UK variant (B.1.1.7), South African variant (B.1.351), and recently the California variant (B.1.427/B.1.429), would increase the public health concean whether they could abrogate the effectivity of the existing SARS-CoV-2 vaccines. Moreover, as part of current social distancing policies, student attendance in academic institutions is restricted in many countries and is being replaced with online classes. However, recent studies have suggested that although children and young adult populations may predominantly exhibit asymptomatic SARS-CoV-2 infections with low pathogenicity, they may still be carriers of infectious viruses^27,28^. In contrast, the majority of patients with severe morbidity and mortality are reportedly elderly people who have limited social activities compared to other age groups^29-31^. It became evident early on that the wide range of SARS-CoV-2 disease severity and the sequelae of infection correlated with the age of the afflicted person and presence of pre-existing medical conditions. Thus, to investigate the differential and diverse clinical manifestations in COVID-19 patients of different ages, COVID-19 age-related disease severity was replicated in ferrets of three different age groups. Although currently there is no apodictic formula to calculate age equivalency between ferrets and humans, various reports indicate that average life span of domesticated ferrets is between 5 and 7 years. However, given their short life span and the observed onset of serious health problems as early as 3-4 years of age in most ferrets, a majority of veterinarians considered ferrets to be geriatric at 3 years of age^32^. Thus, although we cannot accurately correlate considering lifespan and developmental stages of both ferret and human, we believed that ferrets more than 3 years old could represent the aged human population of human.

In this study, we demonstrate the age-associated pathogenesis of SARS-CoV-2 infection using a ferret model. Comparison of clinical symptom values and virus load analysis showed that the aged ferret model fully recapitulates clinical manifestations of COVID-19 in humans (Table 1 and Fig. 1). Recent reports of human patients with SARS-CoV-2 infection in China indicate that aged and comorbid patients carry high virus loads and experience difficulty recovering from severe pneumonia, which leads to high mortality^33^. This finding is well in line with the results of infected ferrets of different ages, although none of the aged ferrets died from SARS-CoV-2 infection in this study. Specifically, aged ferrets showed higher lung damage score, increased virus loads in their respiratory tracts and higher virus shedding compared to the juvenile and young adult ferrets. Moreover, RNAscope in situ hybridization clearly demonstrated higher numbers of SARS-CoV-2 RNA-positive lung cells and infiltrating inflammatory cells in the aged ferret group than in the juvenile and young adult groups, which was ultimately correlated with severe viral pathogenesis in the aged ferret group. While differential ACE2 expression among age groups might affect disease manifestation and virus replication kinetics in ferrets, we found no detectable difference of the ACE2 expression among different age groups. On the other hand, aged ferrets showed high expressions of chemokines (CCL4, CCL5, and CXCL10) and pro-inflammatory cytokines (IFNB1, IFNG, IL1B, IL2, IL6, and IL7) in the early phase of infection.

High expressions of chemokines and pro-inflammatory cytokines may contribute to the aberrant inflammatory response, which in turn causes severe pulmonary pathologies in aged adult ferrets. These findings partially correlate with recent reports of severe COVID-19 cases with increased expression of pro-inflammatory cytokines and chemokines, which are associated with pulmonary inflammation and extensive lung damage^34,35^. Cytokine storm is potentially life-threatening event related to COVID-19. Patients with severe COVID-19 often exhibit acute respiratory distress syndrome, a consequence of cytokine storm resulting from the marked expression of a combination of immune-active molecules. Thus, the pathology of SARS-CoV-2 in infected aged ferrets considerably reflects that of aged COVID-19 patients, making it a valuable animal model to understand the age-dependent viral pathogenesis of SARS-CoV-2. Of note, directly infected ferrets showed the highest viral titer at 2 dpi, which then gradually decreased until 8 (the juvenile group) or 10 dpi (adult and aged ferret groups). However, animals infected through direct contact transmission showed moderate virus titers at 3 dpc which then peaked at 5 dpc in all groups with exception of the juvenile group. This differential phenomenon in infection and transmission groups may be explained by the initial infection dose. The direct infection groups were infected with a high dose of virus (10^6.0^ TCID_50_/mL, which is almost the maximum titer in ferret respiratory tracts) and showed a gradual decrease of virus titer after the maximum titer was reached at 2 dpi. In contrast, ferrets infected by direct contact were presumably infected with lower titers, resulting in virus replication that the maximum titer was reached in naïve animals on 5 dpc. Therefore, the natural infection pattern of SARS-CoV-2 may be more similar to that of the direct contact groups. For example, the juvenile group was presumably exposed to lower amounts of infectious virus which were rapidly cleared in naïve animals.

The role of juveniles (<18 years of age) in the spread of SARS-CoV-2 has not been fully defined. A recent study shows that children are not likely to be the source of SARS-CoV-2 transmission and outbreak, and thus, are minor drivers of the COVID-19 pandemic^20^. In contrast, juveniles typically exhibit the highest rates of influenza virus infection and are considered to play a critical role in influenza virus spread. Thus, it is believed that because juveniles exhibit mild clinical manifestations of COVID-19 they are less likely to transmit infectious SARS-CoV-2 than adults, who exhibit more severe symptoms. Supporting these reports, the present study shows that the infected juvenile ferrets carry low virus titers and are not readily infectious to naïve contact animals. This indicates that besides aged ferrets, juvenile ferrets can also potentially be a useful animal model in understanding the important scientific aspects of the infrequent transmission of SARS-CoV-2 from children to other children and from children to adults.

Although the kinetics of antibody development in SARS-CoV-2 infected-ferrets were comparable among all three groups at 12 dpi, NAb titers were two- and four-fold higher in the young adult and aged ferret groups, respectively, than in the juvenile group (Fig. 3). It is noteworthy that the aged ferret group, which demonstrated more severe clinical symptoms along with the highest viral loads in the respiratory tract, showed a four-fold increase in NAb titer at 21 dpi over that at 12 dpi, suggesting a close association between clinical outcomes and antibody production. This result is also well within agreement with a recent study^35^ reporting that SARS-CoV-2 serum neutralizing antibody levels were higher in severe SARS-CoV-2 patients than in asymptomatic or mild patients. Thus, SARS-CoV-2-infected aged ferrets can also be used to understand the immunological aspects underlying the high neutralizing antibody titer in patients with more severe COVID-19.

Recently, a transcriptome analysis study of COVID-19 patients demonstrated robust induction of type I IFN responses in severe COVID-19 patients compared to mild/moderate COVID-19 patients^25^. Transcriptome analysis during the early stage of SARS-CoV-2 infection in ferrets also revealed enrichment of genes involved in both innate and adaptive immune responses in aged ferrets compared to juvenile and young adult ferrets. Notably, the gene sets related to the type I IFN response and activated M1 macrophages were significantly enriched in SARS-CoV-2-infected aged ferrets at 2 dpi. Furthermore, infected aged ferrets also showed enrichment of genes also expressed in tissues of patients with severe COVID-19, such as lung tissues of post-mortem COVID-19 patients and PBMCs from patients with severe COVID-19 symptoms^25,26^. These results indicate that the SARS-CoV-2-infected aged ferrets not only recapitulate the clinical course of severe symptoms seen in COVID-19 patients, but also the corresponding alteration of transcriptional profiles. In addition, the aged ferrets demonstrated upregulation of genes related to early activation of an adaptive immune response, including T cell and B cell responses, which was maintained up to 5 dpi. These findings may provide a clue to the mechanisms underlying the relatively weak SARS-CoV-2-specific T cell responses and attenuated neutralizing antibody activity observed in asymptomatic or mild COVID-19 patients^37^. Thus, this study provides fundamental information of the *in vivo* gene expression dynamics of the host immune response as seen in hyper-inflammatory responses provoked by severe SARS-CoV-2 infection in COVID-19 patients^26,38^.

Taken together, aged ferrets showed significantly higher virus loads and more severe lung pathology compared to juvenile and young adult ferrets. Moreover, these differences were closely associated with enhanced type I IFN responses and activated M1 macrophages as well as hyper-inflammatory responses in aged ferrets. This aged immune-competent ferret model demonstrates for the first time age-dependent pathogenesis of SARS-CoV-2 infection, making it an invaluable animal model to understand the age-dependency of COVID-19 pathogenesis and the detailed underlying mechanism of asymptomatic infection in juveniles and young adults.

## Methods

### Study design for age-dependent pathogenesis in ferrets

SARS-CoV and SARS-CoV-2 antibody-free female ferrets over 36 months (3 years ≤ Age, n=9), 12-24 months (1 ≤ Age ≤ 2 years, n=9), and under 6 months (Age ≤ 6 months, n=9) were infected through the intranasal (IN) route with the NMC-nCoV02 strain (GISAID accession number: EPI_ISL_1069194) at a dose of 10^5.8^ TCID_50_ per ferret. Nasal washes and fecal specimens were collected every day for 10 days from each group of ferrets to measure viral titers. To measure the infectious live virus in the collected specimens, each sample was inoculated with Vero cells, which were then incubated for 4 days prior to virus isolation. To assess viral replication in various organs of ferrets following SARS-CoV-2 infection, ferrets (n=3/group) were sacrificed at 3 and 5 dpi to harvest their nasal turbinates and lung tissues with individual scissors to avoid cross-contamination. The left lung lobes from the harvested whole lungs were homogenized for virus titration in Vero cells and the right lung lobes were immediately fixed in 10% neutral-buffered formalin solution for further histopathological examinations.

### Quantitative real-time RT-PCR (qRT-PCR) to detect SARS-CoV-2 RNA

To measure the viral titer in respiratory and gastrointestinal tracts, nasal washes and rectal swab samples collected from ferrets were suspended in cold phosphate-buffered saline (PBS) containing antibiotics (5% penicillin/streptomycin; Gibco). To measure the viral copy number, total RNA was extracted from the collected samples using RNeasy Mini® kit (QIAGEN, Hilden, Germany) according to the manufacturer's instructions. A cDNA synthesis kit (Omniscript Reverse Transcriptase; QIAGEN, Hilden, Germany) was used to synthesize single-strand cDNA from total viral RNA. To quantify viral RNA copy number, quantitative real-time RT-PCR (qRT-PCR) was performed for the partial E gene primer set: forward primer, SARS-CoV-2-E-forward, atgtactcattcgtttcggaagag; and reverse primer, SARS-CoV-2-E-reverse, ctagagttcctgatcttctggtctaa with the SYBR Green kit (iQTM SYBR Green supermix kit, Bio-Rad, Hercules, CA, USA). The number of viral RNA copies was calculated and compared to the number of copies of the standard control.

### SARS-CoV-2 isolation

SARS-CoV-2 was isolated from serial nasal wash specimens of each ferret group by inoculating with Vero cells in DMEM (Sigma Aldrich) supplemented with 2% fetal bovine serum (Fisher Scientific), 1 mM L-glutamine (Thermo Fischer), 50 U/mL penicillin (Thermo Fischer), and 50 μg/mL streptomycin (Thermo Fischer). Briefly, specimens were centrifuged at 4°C at 1200 rpm for 15 mins and the supernatants were incubated with Vero cells for 2 hours. Media (DMEM) was changed daily and cells were monitored for 4 days to examine the cytopathic effects (CPEs). To confirm virus isolation, we performed qRT-PCR on supernatants from infected cell cultures using S gene-specific primer sets [Forward (5’-3’): AGGGCAAACTGGAAAGATTGCTGA, Reverse (5’-3’): TCTGTG CAGTTAACATCCTGATAAAGAAC].

### RNA-Sequencing

Total RNA was isolated using TRIzol reagent (Invitrogen) according to the manufacturer’s instructions. RNA quality was assessed with Agilent 2100 Bioanalyzer using an RNA 6000 Nano Chip (Agilent Technologies), and RNA was quantified using an ND-2000 Spectrophotometer (Thermo Fisher Scientific). Extracted RNAs were processed using the TruSeq Stranded mRNA Sample Prep Kit (Illumina) according to the manufacturer’s instructions. High-throughput sequencing was performed as paired-end 150 sequencing runs using NovaSeq 6000 (Illumina). Raw reads were assembled and low-quality reads were filtered using Cutadapt (version 2.8). Filtered reads were aligned on a reference genome downloaded from Ensembl (MusPutFur1.0, Accession number: GCF_000215625.1) using STAR (version 2.7.1a) and annotated with additive human ortholog genes from the human reference database (Biomart database, GRCh38). Gene counts were normalized to valid library size and the dimensional reduction was performed by principal components analysis (PCA) using the top 2 principal components (PCs) throughout the whole samples. Two-sided Wald test was performed to analyze the differentially expressed genes (DEGs) according to each condition with DESeq2 (version 1.26.0)^39^. DEGs were determined according to cutoffs of a *p*-value < 0.05 and a log2 fold change > 0.5 for by day and > 1 for by age. To analyze functional profiles of each condition, gene set enrichment analysis (GSEA) was performed in Gene ontology: Biological process database (GO. BP) and specific public gene set using clusterProfiler (version 3.14.3)^40-42^. To compare the gene set enrichment score for the specific gene sets, gene set variation analysis (GSVA) (version 1.34.0) was performed^43^. To calculate the severe COVID-19 signature score, significantly upregulated genes in severe COVID-19 patients were used^25,26^.

### RNAscope *in situ* hybridization and pathology

SARS-CoV-2 RNA (Spike gene) and ACE2 were detected using the Spike-specific and ACE2 probe (Advanced Cell Diagnostics, Cat. # 848561, # 848151) and visualized using RNAscope 2.5 HD Reagent Kit RED (Advanced Cell Diagnostics, Cat. # 322360). Lung tissue sections were fixed in 10% neutral-buffered formalin and embedded in paraffin, according to the manufacturer’s instructions, followed by counterstaining with Gill’s hematoxylin #1 (Polysciences, cat # 24242-1000). For pathological examination, the embedded tissues were sectioned and dried for three days at room temperature. Histopathological examination was conducted by hematoxylin and eosin (H&E) staining. Slides were viewed using Olympus IX 71 (Olympus, Tokyo, Japan) microscope with DP controller software to capture images.

### Histopathology scoring analysis

Quantification of SARS-CoV-2 RNA positive- and ACE2 positive cells. From 400x magnification area, all positively stained cells (SARS-CoV-2 RNA positive- and ACE2 positive) were counted in triplicate and the average calculated. Ferret lungs were graded depending on the degree of inflammation observed in each individual lung at 3 and 5 dpi.

### Serologic assay

The neutralizing antibody (NAb) assay against SARS-CoV-2 was carried out using a micro-neutralization assay in Vero cells. Collected ferret serum specimens were inactivated at 56°C for 30 min. Initial 1:2 serum dilutions were made with the medium, and two-fold serial dilutions of all samples were made to a final serum dilution of 1:2 to 1:1024. For each well, 50 μL of serially diluted serum was mixed with 50 μL (equal volume) of 100 TCID_50_ of SARS-CoV-2 and incubated at 37 °C for 1 h to neutralize the infectious virus. The mixtures were then transferred to the Vero cell monolayers. Vero cells were incubated at 37°C in 5% CO_2_ for 4 days and monitored for 50% reduction in cytopathic effect (CPE).

### ELISA (Enzyme-linked immunosorbent assay)

Anti-SARS-CoV-2 IgG ELISA for ferret serum (cat. EH4397, FineTest, Wuhan, China) targeting Nucleocapsid (N) protein were run according to the manufacturer’s protocol. Briefly, serum was diluted 1:50 in each well with the provided sample buffer and then incubated at 37 °C for 30 min. Sample wells were washed three times with a provided wash buffer before the provided HRP-labeled antibody working solution was added (0.05 mL/well) and incubated at 37 °C for 30 min. After a second wash step, the provided TMB substrate solution was added (0.05 mL/well) and incubated at ambient temperature for 15 min. The provided stop solution was then added (0.05 mL/well) and the absorbance of sample wells was measured as optical density (O.D.) with a spectrometer (iMark Microplate Reader; Bio-Rad) at 450 nm.

## Supplementary Material

Supplement

## Figures and Tables

**Table 1.** Clinical scores of ferrets infected with SARS-CoV-2

Group		0dpi	2dpi	4dpi	6dpi	8dpi	10dpi
**G1 (Juvenile)**	Cough	0.00	0.00	0.00	0.00	0.00	0.00
	Runny nose	0.00	0.56±0.50	0.33±0.47	0.00	0.00	0.00
	Movement, activity	0.00	0.44±0.50	0.17±0.37	0.00	0.00	0.00
	Total	0.00	1.00	0.50	0.00	0.00	0.00
**G2 (Young adult)**	Cough	0.00	0.33±0.47	1.00	0.83±0.37	0.00	0.00
	Runny nose	0.00	0.22±0.42	1.33±0.47	1.17±0.37	0.00	0.00
	Movement, activity	0.00	0.67±0.67	1.83±0.37	1.83±0.37	1.00	0.00
	Total	0.00	1.22	4.17	3.83	1.00	0.00
**G3 (Aged adult)**	Cough	0.00	0.56±0.50	1.00	1.00	0.00	0.00
	Runny nose	0.00	0.44±0.68	1.67±0.47	1.17±0.37	0.67±0.47	0.33±0.47
	Movement, activity	0.00	0.78±0.92	2.00	1.83±0.37	1.00	0.67±0.47
	Total	0.00	1.78	4.67	4.00	1.67	1.00

Observational clinical symptoms: Cough, rhinorrhea, movement, and activity. Score: 0; normal, 1: occasional, mild reduced activity, 2: frequent, reduced activity.

* Scores were measured by observation of clinical symptoms for at least 20 minutes in each group of ferrets based on the following criteria: Cough: 0; no evidence of cough, 1; occasional cough, 2; frequent cough (score 2).

Rhinorrhea: 0; no nasal rattling or sneezing, 1; moderate nasal discharge on external nares, 2; severe nasal discharge on external nares.

Movement, activity: 0; normal movement and activity, 1; mild reduced movement and activity, 2; evidence of reduced movement and activity.
