# Supplementary material for: Age-dependent pathogenic characteristics of SARS-CoV-2 infection in ferrets
Source: Res Sq. 2021 Mar 29:rs.3.rs-131380. Preprint. [Version 2] doi: 10.21203/rs.3.rs-131380/v2 (PMC8020987; doi:10.21203/rs.3.rs-131380/v2)
Supplement: Supplement [file 4eff15bc94c1f07a864bdf5d.pdf]

Supplementary information for

Age-dependent pathogenic characteristics of SARS-CoV-2 infection in ferrets

Kim et al.

Supplementary Fig. 1 – 6

Supplementary Table 1 - 4

Fig. S1

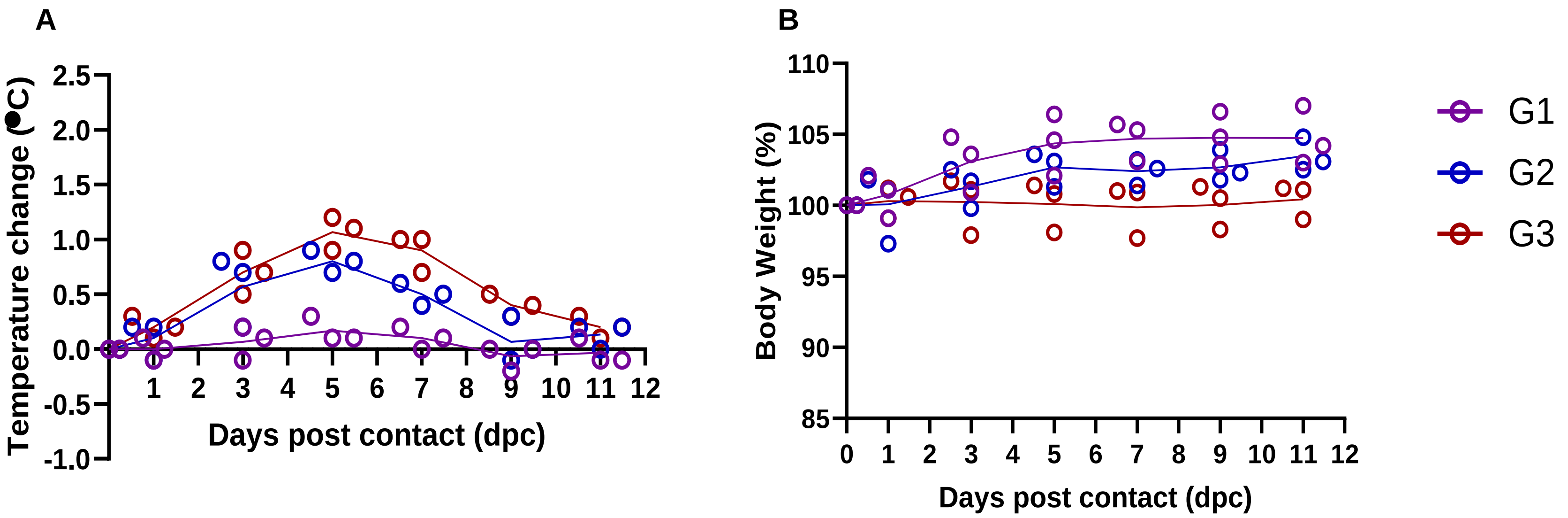
A
Temperature change (●C)
2.5
2.0
1.5
1.0
0.5
0.0
-0.5
-1.0
1 2 3 4 5 6 7 8 9 10 11 12
Days post contact (dpc)
B
Body Weight (%)
110
105
100
95
90
85
0 1 2 3 4 5 6 7 8 9 10 11 12
Days post contact (dpc)
G1
G2
G3

Supplementary Fig. 1. Change in body temperature and weight of direct contact ferrets. Temperature changes and relative body weight were measured in direct contact transmission ferrets from each different age group. Temperature is represented as ℃ and weight is demonstrated as a percentage of the initial body weight.

Fig. S2

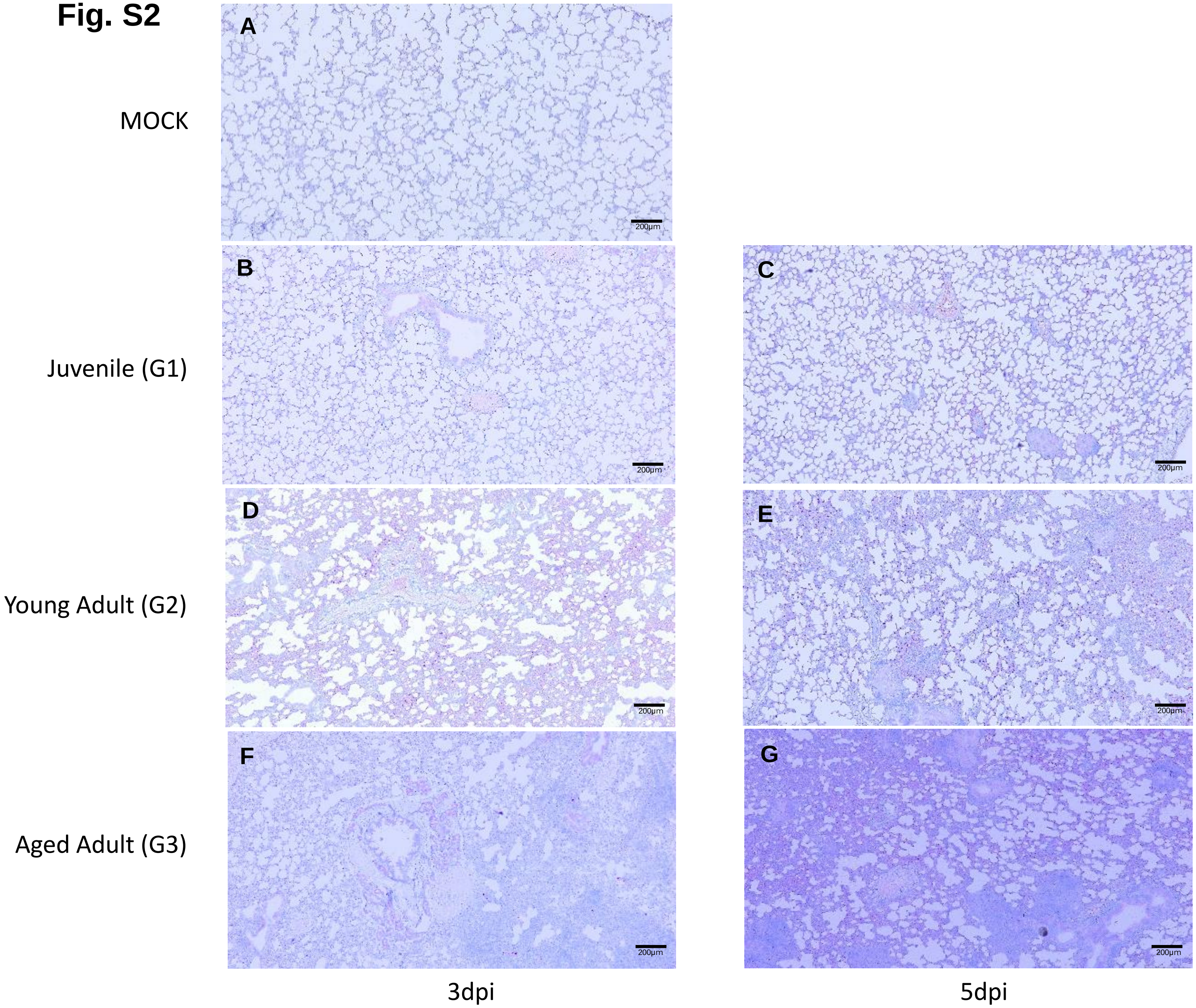
A
B
C
D
E
F
G
200µm
MOCK
Juvenile (G1)
Young Adult (G2)
Aged Adult (G3)
3dpi
5dpi

Supplementary Fig. 2. *In situ* lung analysis and histopathology of lungs from SARS-CoV-2 infected ferret groups. Ferrets were inoculated with $10^{5.8}$ $TCID_{50}$ of NMC-nCoV02 virus. Lung tissues were harvested on days 3 (B, D, and F) and 5 (C, E, and G) post-inoculation. Lung regions were compared by histopathology among the different age groups of ferrets: (A) mock infected, (B-C) juveniles (less than 6 months, G1 group), (D-E) young adults (1 to 2 years, G2 group), and (F-G) aged ferrets (older than 3 years). Magnification x40 and scale bar 200 μm.

Fig. S3

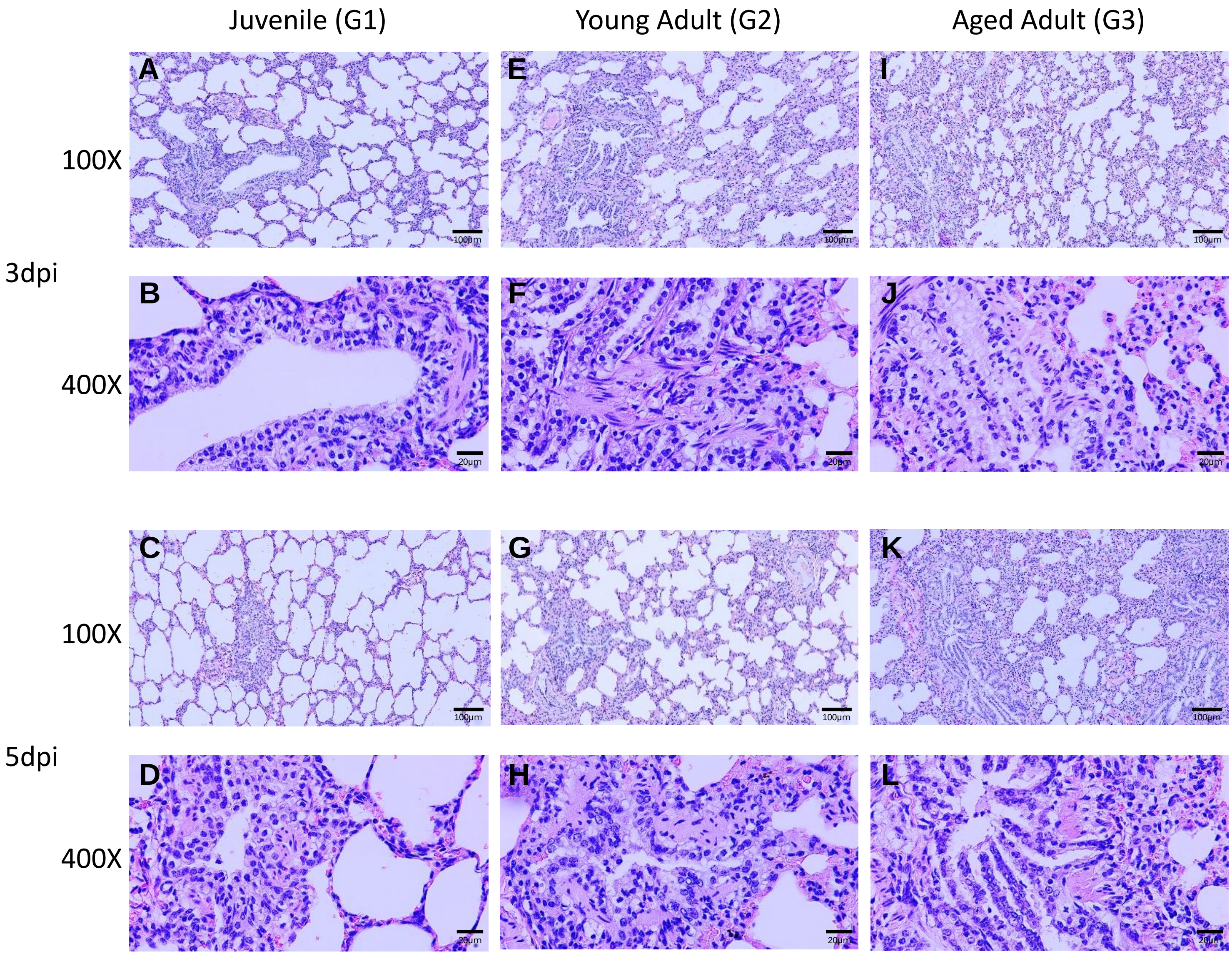
Juvenile (G1)
Young Adult (G2)
Aged Adult (G3)
3dpi
5dpi
100X
400X
100X
400X
A
B
C
D
E
F
G
H
I
J
K
L
100μm
20μm

Supplementary Fig. 3. Histopathology of lungs from SARS-CoV-2 infected ferret groups. Ferrets were inoculated with $10^{5.8}$ $TCID_{50}$ of NMC-nCoV02 virus. Lung tissues were harvested on days 3 and 5 post-inoculation. Histopathological lung regions were compared among the different age groups of ferrets: (A-D) juvenile (less than 6 months, G1 group), (E-H) young adults (1 to 2 years, G2 group), and (I-L) aged ferrets (older than 3 years). Histopathological observations indicated that moderate interstitial pneumonia with thickened alveolar septa (A, C, E, G, I, and K, magnification 100x and scale bar 100µm). G3 group showed more severe lung damage and infiltration of lymphocytes compared G1 and G3 gorups (B, D, F, H, J, and L, magnification 400x and scale bar 20µm).

Fig. S4

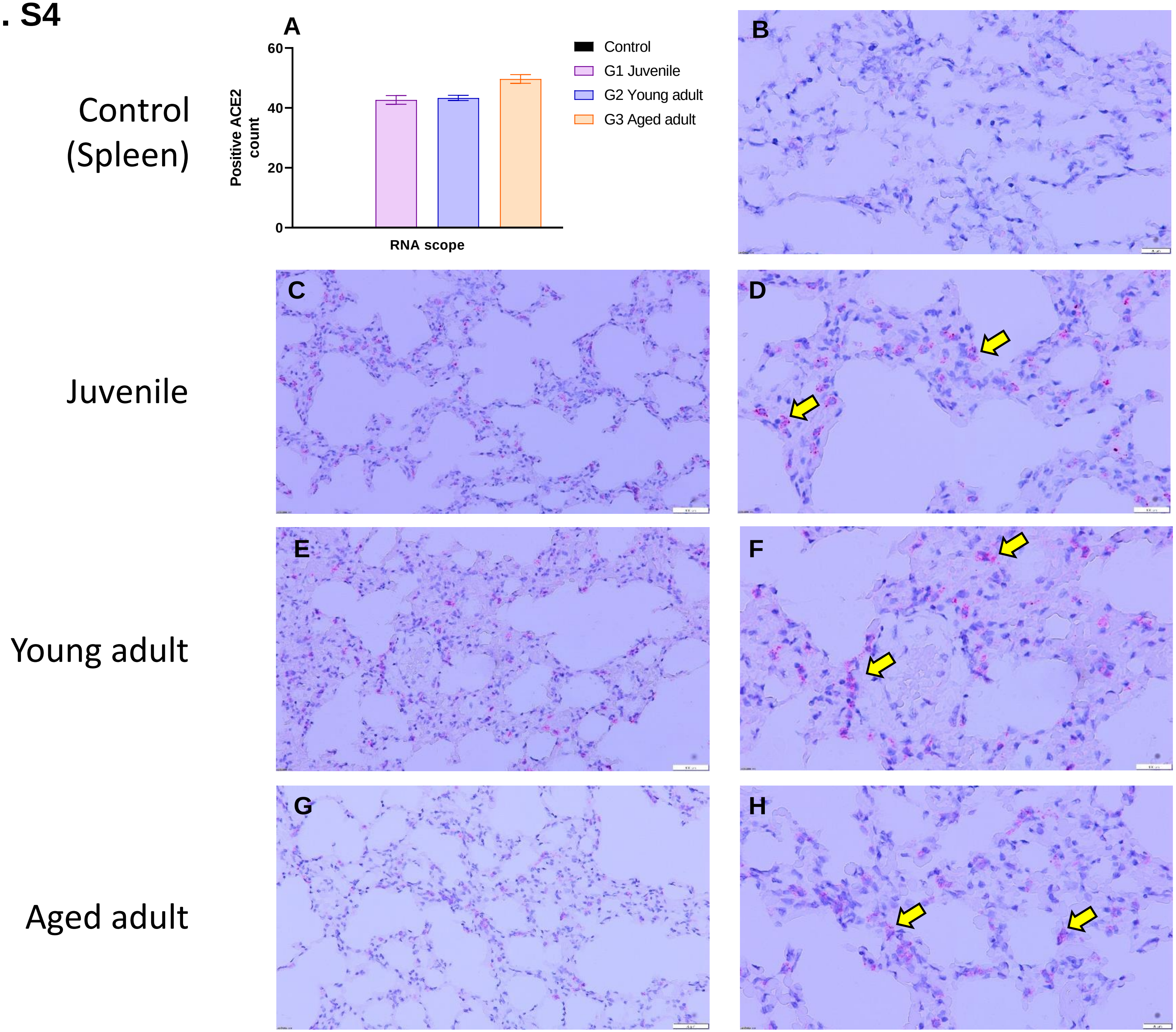
Control
(Spleen)
Juvenile
Young adult
Aged adult
A
Positive ACE2 count
60
40
20
0
RNA scope
Control
G1 Juvenile
G2 Young adult
G3 Aged adult
B
C
D
E
F
G
H

Supplementary Fig. 4. RNAscope *in situ* hybridization of ferret ACE2 receptor expression in lung tissues. To quantitate ACE2 expression in ferret lungs, RNAscope *in situ* hybridization was performed using an ACE2 probe (Advanced Cell Diagnostics, cat. #848151) and visualized using RNAscope 2.5 HD Reagent Kit RED (Advanced Cell Diagnostics, cat. #322360). Positive ACE2 cumulative number from ACE2 stained lung cells in each slide (using 400x magnification) (A). ACE2-positive cells (Yellow arrows) in lung tissues of control (B), juvenile (≤ 6 months, G1 group) (C-D), young adult (1 ≤ age ≤ 2 years, G2 group) (E-F), and aged ferrets (3-year ≤ ages) (G-H). Magnification 200x (A, C, E, and G). Magnification 400x (B, D, F, and H).

Fig. S5

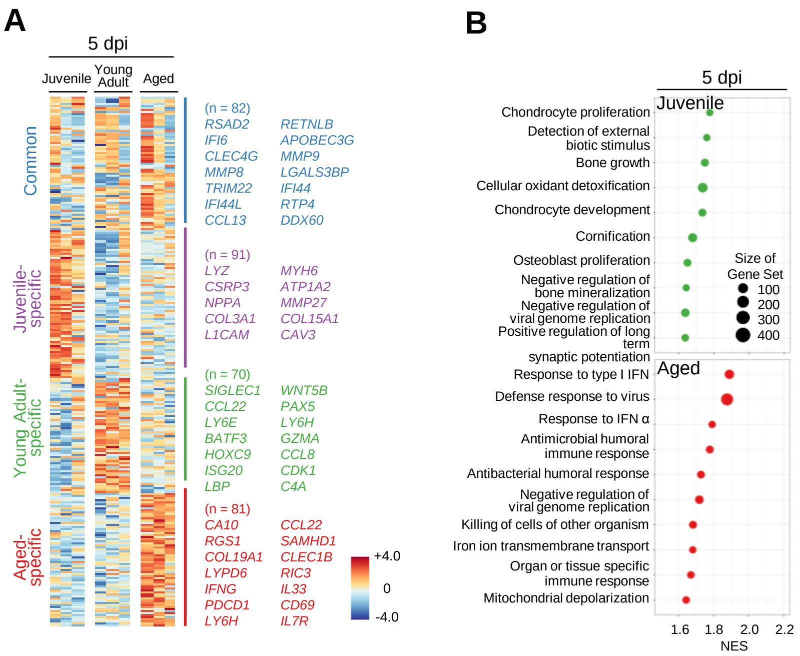

A
5 dpi
Juvenile
Young Adult
Aged
Common
Juvenile-specific
Young Adult-specific
Aged-specific
(n = 82)
RSAD2
IFI6
CLEC4G
MMP8
TRIM22
IFI44L
CCL13
RETNLB
APOBEC3G
MMP9
LGALS3BP
IFI44
RTP4
DDX60
(n = 91)
LYZ
CSRP3
NPPA
COL3A1
L1CAM
MYH6
ATP1A2
MMP27
COL15A1
CAV3
(n = 70)
SIGLEC1
CCL22
LY6E
BATF3
HOXC9
ISG20
LBP
WNT5B
PAX5
LY6H
GZMA
CCL8
CDK1
C4A
(n = 81)
CA10
RGS1
COL19A1
LYPD6
IFNG
PDCD1
LY6H
CCL22
SAMHD1
CLEC1B
RIC3
IL33
CD69
IL7R
+4.0
0
-4.0
B
5 dpi
Juvenile
Chondrocyte proliferation
Detection of external biotic stimulus
Bone growth
Cellular oxidant detoxification
Chondrocyte development
Cornification
Osteoblast proliferation
Negative regulation of bone mineralization
Negative regulation of viral genome replication
Positive regulation of long term synaptic potentiation
Size of Gene Set
100
200
300
400
Aged
Response to type I IFN
Defense response to virus
Response to IFN α
Antimicrobial humoral immune response
Antibacterial humoral response
Negative regulation of viral genome replication
Killing of cells of other organism
Iron ion transmembrane transport
Organ or tissue specific immune response
Mitochondrial depolarization
1.6
1.8
2.0
2.2
NES

Supplementary Fig. 5. Heatmap of age-specific differentially expressed genes (DEGs), compared to control at 5 dpi shows that genes related to tissue repair and T cell activation were upregulated in 5 dpi (A). The ‘Common’ gene sets were composed of genes differentially upregulated in more than two groups, while ‘Juvenile-specific’, ‘Young adult-specific’ and ‘Aged-specific’ gene sets were composed of genes uniquely upregulated in each group. Representative immune-related genes were listed next to the heatmap. Bar plots showing normalized enrichment score (NES) from enrichment analysis of representative GO biological pathway at 5 dpi (B).

Fig. S6

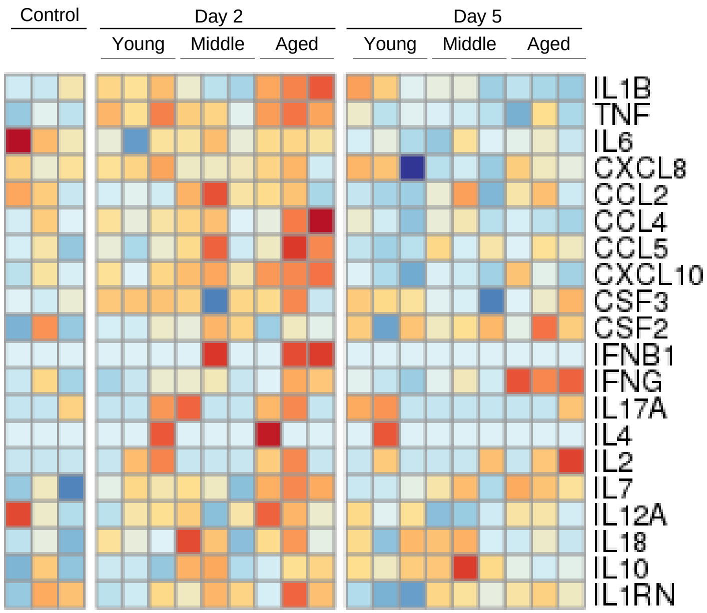

	D0 vs D2	D0 vs D5	D2 vs D5
IL1B	*		*
TNF	***		***
IL6	***	***	
CXCL8			
CCL2			
CCL4	**		**
CCL5	**		***
CXCL10	***		***
CSF3			
CSF2			
IFNB1			
IFNG		*	*
IL17A			
IL4			
IL2			
IL7	*		
IL12A			
IL18	*		
IL10			
IL1RN			

Supplementary Fig. 6. Expression of various cytokines and chemokines in lung tissues. The heatmap shows the induction of cytokines and chemokines as measured via RNA sequencing in aged, juvenile, and young adult ferrets at two different time points (2 and 5 dpi). The color gradient represents expression levels. Asterisks indicate statistical significance compared with control (MOCK) as evaluated by two way ANOVA Tukey's multiple comparisons tests (* indicates $p < 0.05$, ** indicates $p < 0.005$, and *** indicates $p < 0.0005$).

Table S1. Clinical scores of individual ferrets infected with SARS-CoV-2

Group		0dpi	2dpi	4dpi	6dpi	8dpi	10dpi
G1 (Juvenile)	Cough	0.00	0.00	0.00	0.00	0.00	0.00
	Runny nose	0.00	0.00	0.00	0.00	0.00	0.00
	Movement, activity	0.00	0.00	0.00	0.00	0.00	0.00
	Cough	0.00	0.00	0.00	0.00	0.00	0.00
	Runny nose	0.00	0.00	1.00	0.00	0.00	0.00
	Movement, activity	0.00	0.00	0.00	0.00	0.00	0.00
	Cough	0.00	0.00	0.00	0.00		
	Runny nose	0.00	1.00	0.00	0.00		
	Movement, activity	0.00	0.00	0.00	0.00		
	Cough	0.00	0.00	0.00	0.00		
	Runny nose	0.00	1.00	0.00	0.00		
	Movement, activity	0.00	0.00	0.00	0.00		
	Cough	0.00	0.00				
	Runny nose	0.00	1.00				
	Movement, activity	0.00	1.00				
	Cough	0.00	0.00	0.00	0.00		
	Runny nose	0.00	0.00	0.00	0.00		
	Movement, activity	0.00	0.00	1.00	0.00		
	Cough	0.00	0.00				
	Runny nose	0.00	1.00				
	Movement, activity	0.00	1.00				
	Cough	0.00	0.00	0.00	0.00	0.00	0.00
	Runny nose	0.00	0.00	1.00	0.00	0.00	0.00
	Movement, activity	0.00	1.00	0.00	0.00	0.00	0.00
	Cough	0.00	0.00				
	Runny nose	0.00	1.00				
	Movement, activity	0.00	1.00				
Average	Cough	0.00	0.00	0.00	0.00	0.00	0.00
	Runny nose	0.00	0.56±0.50	0.33±0.47	0.00	0.00	0.00
	Movement, activity	0.00	0.44±0.50	0.17±0.37	0.00	0.00	0.00
G2 (Young adult)	Cough	0.00	0.00				
	Runny nose	0.00	1.00				
	Movement, activity	0.00	1.00				
	Cough	0.00	1.00	1.00	1.00		
	Runny nose	0.00	0.00	2.00	2.00		
	Movement, activity	0.00	0.00	2.00	2.00		
	Cough	0.00	1.00				
	Runny nose	0.00	0.00				
	Movement, activity	0.00	1.00				
	Cough	0.00	1.00				
	Runny nose	0.00	1.00				
	Movement, activity	0.00	2.00				
	Cough	0.00	0.00	1.00	1.00		

	Runny nose	0.00	0.00	1.00	1.00		
	Movement, activity	0.00	1.00	2.00	2.00		
	Cough	0.00	0.00	1.00	0.00		
	Runny nose	0.00	0.00	1.00	1.00		
	Movement, activity	0.00	1.00	2.00	1.00		
	Cough	0.00	0.00	1.00	1.00	0.00	0.00
	Runny nose	0.00	0.00	2.00	1.00	0.00	0.00
	Movement, activity	0.00	0.00	1.00	2.00	1.00	0.00
	Cough	0.00	0.00	1.00	1.00	0.00	0.00
	Runny nose	0.00	0.00	1.00	1.00	0.00	0.00
	Movement, activity	0.00	0.00	2.00	2.00	1.00	0.00
	Cough	0.00	0.00	1.00	1.00	0.00	0.00
	Runny nose	0.00	0.00	1.00	1.00	0.00	0.00
	Movement, activity	0.00	0.00	2.00	2.00	1.00	0.00
**Average**	Cough	0.00	0.33±0.47	1.00	0.83±0.37	0.00	0.00
	Runny nose	0.00	0.22±0.42	1.33±0.47	1.17±0.37	0.00	0.00
	Movement, activity	0.00	0.67±0.67	1.83±0.37	1.83±0.37	1.00	0.00
**G3 (Aged adult)**	Cough	0.00	1.00	1.00	1.00	0.00	0.00
	Runny nose	0.00	0.00	2.00	2.00	1.00	0.00
	Movement, activity	0.00	0.00	2.00	1.00	1.00	0.00
	Cough	0.00	1.00	1.00	1.00		
	Runny nose	0.00	0.00	2.00	1.00		
	Movement, activity	0.00	1.00	2.00	2.00		
	Cough	0.00	0.00	1.00	1.00		
	Runny nose	0.00	0.00	2.00	1.00		
	Movement, activity	0.00	0.00	2.00	2.00		
	Cough	0.00	1.00				
	Runny nose	0.00	1.00				
	Movement, activity	0.00	2.00				
	Cough	0.00	1.00				
	Runny nose	0.00	1.00				
	Movement, activity	0.00	2.00				
	Cough	0.00	0.00	1.00	1.00		
	Runny nose	0.00	0.00	1.00	1.00		
	Movement, activity	0.00	0.00	2.00	2.00		
	Cough	0.00	0.00	1.00	1.00	0.00	0.00
	Runny nose	0.00	0.00	2.00	1.00	0.00	0.00
	Movement, activity	0.00	0.00	2.00	2.00	1.00	1.00
	Cough	0.00	0.00	1.00	1.00	0.00	0.00
	Runny nose	0.00	0.00	1.00	1.00	1.00	1.00
	Movement, activity	0.00	0.00	2.00	2.00	1.00	1.00
	Cough	0.00	1.00				
	Runny nose	0.00	2.00				
	Movement, activity	0.00	2.00				
**Average**	Cough	0.00	0.56±0.50	1.00	1.00	0.00	0.00

Runny nose	0.00	0.44±0.68	1.67±0.47	1.17±0.37	0.67±0.47	0.33±0.47
Movement, activity	0.00	0.78±0.92	2.00	1.83±0.37	1.00	0.67±0.47

Observational clinical symptoms: Cough, rhinorrhea, movement, and activity.
Score: 0; normal, 1: occasional, mild reduced activity, 2: frequent, reduced activity.
* Scores were measured by observation of clinical symptoms for at least 20 minutes in each group of ferrets based on the following criteria: Cough: 0; no evidence of cough, 1; occasional cough, 2; frequent cough (score 2).
Rhinorrhea: 0; no nasal rattling or sneezing, 1; moderate nasal discharge on external nares, 2; severe nasal discharge on external nares.
Movement, activity: 0; normal movement and activity, 1; mild reduced movement and activity, 2; evidence of reduced movement and activity.

Table S2. Clinical symptom score in contact group ferrets.

Group		0dpc	1dpc	3dpc	5dpc	7dpc	9dpc	11dpc
**G1 (Juvenile)**	Cough	0.00	0.00	0.00	0.00	0.00	0.00	0.00
	Runny nose	0.00	0.00	0.00	0.00	0.00	0.00	0.00
	Movement, activity	0.00	0.00	0.00	0.00	0.00	0.00	0.00
	Cough	0.00	0.00	0.00	0.00	0.00	0.00	0.00
	Runny nose	0.00	0.00	0.00	0.00	0.00	0.00	0.00
	Movement, activity	0.00	0.00	0.00	0.00	0.00	0.00	0.00
	Cough	0.00	0.00	0.00	0.00	0.00	0.00	0.00
	Runny nose	0.00	0.00	0.00	0.00	0.00	0.00	0.00
	Movement, activity	0.00	0.00	0.00	0.00	0.00	0.00	0.00
**Average**	Cough	0.00	0.00	0.00	0.00	0.00	0.00	0.00
	Runny nose	0.00	0.00	0.00	0.00	0.00	0.00	0.00
	Movement, activity	0.00	0.00	0.00	0.00	0.00	0.00	0.00
**G2 (Young adult)**	Cough	0.00	0.00	0.00	0.00	0.00	0.00	0.00
	Runny nose	0.00	0.00	1.00	1.00	0.00	0.00	0.00
	Movement, activity	0.00	0.00	1.00	0.00	0.00	0.00	0.00
	Cough	0.00	0.00	0.00	0.00	0.00	0.00	0.00
	Runny nose	0.00	0.00	1.00	0.00	0.00	0.00	0.00
	Movement, activity	0.00	0.00	1.00	1.00	0.00	0.00	0.00
	Cough	0.00	0.00	0.00	0.00	0.00	0.00	0.00
	Runny nose	0.00	0.00	0.00	0.00	0.00	0.00	0.00
	Movement, activity	0.00	0.00	1.00	0.00	0.00	0.00	0.00
**Average**	Cough	0.00	0.00	0.00	0.00	0.00	0.00	0.00
	Runny nose	0.00	0.00	0.67±0.47	0.33±0.47	0.00	0.00	0.00
	Movement, activity	0.00	0.00	1.00	0.33±0.47	0.00	0.00	0.00
**G3 (Aged adult)**	Cough	0.00	0.00	0.00	0.00	0.00	0.00	0.00
	Runny nose	0.00	0.00	1.00	1.00	1.00	0.00	0.00
	Movement, activity	0.00	0.00	1.00	2.00	1.00	0.00	0.00
	Cough	0.00	0.00	0.00	0.00	0.00	0.00	0.00
	Runny nose	0.00	0.00	1.00	1.00	0.00	0.00	0.00
	Movement, activity	0.00	0.00	1.00	1.00	1.00	0.00	0.00
	Cough	0.00	0.00	0.00	0.00	0.00	0.00	0.00
	Runny nose	0.00	0.00	1.00	2.00	1.00	0.00	0.00
	Movement, activity	0.00	0.00	1.00	1.00	1.00	0.00	0.00
**Average**	Cough	0.00	0.00	0.00	0.00	0.00	0.00	0.00
	Runny nose	0.00	0.00	1.00	1.33±0.47	0.67±0.47	0.00	0.00
	Movement, activity	0.00	0.00	1.00	1.33±0.47	1.00	0.00	0.00

Observational clinical symptoms: Cough, rhinorrhea, movement, and activity.

Score: 0; normal, 1: occasional, mild reduced activity, 2: frequent, reduced activity.

* Scores were measured by observation of clinical symptoms for at least 20 minutes in each group of ferrets based on the following criteria: Cough: 0; no evidence of cough, 1; occasional cough, 2; frequent cough (score 2).

Rhinorrhea: 0; no nasal rattling or sneezing, 1; moderate nasal discharge on external nares, 2; severe nasal discharge on external nares.
Movement, activity: 0; normal movement and activity, 1; mild reduced movement and activity, 2; evidence of reduced movement and activity.

Table S3. Genes used In principal component analysis (PCA)

PC1	PC2
CLCA1	*RSAD2*
DNAH9	*ISG15*
MUC5AC	*MX1*
BPIFB1	*IFIT3*
HBB	*CMPK2*
CDHR4	*DHX58*
ERICH3	*OAS3*
CFAP43	*IFI6*
ECT2L	*USP18*
HBA2	*MX2*
DNAH5	*OAS1*
LPO	*OASL*
DNAH10	*APOBEC3G*
CFAP65	*HERC6*
DNAH1	*UBA7*
ERN2	*DDX58*
TMC5	*IFIT2*
VMO1	*IRF7*
CAPS	*RNF213*
MAPK15	*DDX60*
AGR2	*SAMD9L*
RSPH1	*GBP1*
EML6	*IFIH1*
GP2	*HERC5*
BPIFA1	*NLRC5*
LAYN	*CXCL10*
CPA3	IFI44
DRC7	CXCL11
FOXJ1	IFI44L
DNAH11	SAMHD1
DNAI2	PARP14
DNAH7	BATF2
SPAG6	LGALS9C
ACE	UBE2L6
HYDIN	TRANK1
MUC13	STAT1
CCDC114	TRIM6-TRIM34
TFF3	EPSTI1
AK7	DTX3L
SCGB3A1	C3
CCDC180	LGALS3BP
CFAP52	TRIM22
LRRC43	ZNFX1
TTLL10	BST2
NME9	PYHIN1
TTC21A	C4A
VWA3A	NT5C3A
CCDC146	PARP12
STMND1	PARP15
CCDC40	ZBP1
UBXN10	OAS2
WDR63	EIF2AK2
CFAP45	PML
COL15A1	ETV7

FAM216B	NUP153
DNAAF1	AEBP1
SLC44A4	HSH2D
C17orf97	HELZ2
WDR38	PARP9
COL1A1	IL4I1
CSF3R	IFIT5
DNAH6	HLA-DQA1
VWA3B	PARM1
CAPSL	AC104389.5
WDR66	PRG4
ANKMY1	STAT2
CFAP46	C19orf66
PLEKHS1	IGHA2
ZCCHC18	APOL5
SNTN	CHIT1
CFAP126	SIGLEC1
CDHR3	SP100
APOA1	PLSCR1
FRMPD2	TRIM25
ALAS2	ERAP2
DCDC2	TDRD7
ACOD1	RTP4
TMEM2	
COL3A1	
CFAP99	
FAM183A	
CATIP	
CFAP157	
LRRC23	
TOGARAM2	
ALS2CR12	
PTN	
AC010255.3	
MAP3K19	
THBD	

Table S4. A list of differentially expressed genes (DEGs)

[at 2 dpi]

Common	Juvenile	Young Adult	Aged
ZBP1	*CCNJL*	*RARRES1*	*CA10*
MX1	*EPHA5*	*ESCO2*	*DNTT*
RSAD2	*BTNL9*	*CCL22*	*MUC5AC*
MX2	*CAV3*	*DDIAS*	*ISLR2*
EBF2	*GPX6*	*TMEM255B*	*KIAA1024*
BTNL2	*OSM*	*WNT5B*	*TTLL8*
ACOD1	*SORCS1*	*C10ORF99*	*BST2*
SHISA3	*LYZ*	*PAX5*	*ZNFX1*
TMEM81	*L1CAM*	*RBP5*	*FSHR*
LGALS9C	*SLC5A7*	*TMIGD2*	*TLR7*
PML	*GLTPD2*	*SIGLEC1*	*CLEC4F*
CXCL10	*EEF1A2*	*PAX8*	*MS4A2*
IFI44L	*COL3A1*	*ESPL1*	*HIST1H4E*
SLFN14	*LRIT3*	*LGSN*	*RIPOR3*
CXCL11	*MGAM2*	*FXYD2*	*TENM2*
MYL7	*CILP*	*LY6E*	*ST6GALNAC1*
FAP	*COL5A3*	*CDK1*	*CSF3R*
CLEC4G	*APLNR*	*MXRA5*	*CRYBG2*
CD177	*TFF2*	*KCNK2*	*C3AR1*
TELOMERASE-VERT	*MYH6*	*SCG3*	*TRAV21*
TUBB1	*SNAP25*	*LY6H*	*KRT24*
TRANK1	*IGHV7-4-1*	*ACOT6*	*VWCE*
NUGGC	*ITIH3*	*CDKN3*	*NLRC5*
TNF	*TNFAIP6*	*HIST1H4G*	*EPX*
IFI6	*NRK*	*SHOX2*	*XKRX*
SAMD9L	*LMOD2*	*SKA1*	*ACP7*
UBE2L6	*KRT23*	*CRABP2*	*S100A12*
ASB16	*ATP1A2*	*SECTM1*	*BATF2*
HERC6	*ADAMTS17*	*NOXO1*	*PIANP*
HERC5	*COMP*	*NCAPG*	*TTC36*
CAPN9	*CSRP3*	*C12ORF40*	*EN1*
LY6G6C	*ZSCAN10*	*C4A*	*KIAA1683*
GBP1	*DHRS7C*	*TRIM9*	*LMTK3*
HES4	*UCP3*	*CREB3L1*	*OMG*
EPSTI1	*ADORA1*	*TCTEX1D1*	*KIR3DL3*
CPA3	*HMCN2*	*LBP*	*GPR142*
FER1L6	*AL662899.1*	*ADAD1*	*CHIT1*
APOBEC3G	*CD300LG*	*OTX2*	*TREML1*
DDX58	*C10ORF90*	*MB21D1*	*ADGRF1*
LVRN	*ST18*	*NOTO*	*TCN1*
EPHX4	*SYT4*	*CPA4*	*PADI4*
ISG15	*CPA1*	*BATF3*	*IL17F*
PARP15	*TNNC1*	*GZMA*	*CDH17*
IFI44	*PLA2G4D*	*LY86*	*LTF*
PARP14	*TDRD9*	*TVP23A*	*RHAG*
BPIFB1	*ANK1*	*HTR3A*	*CHST4*
WBSCR17	*GJB1*	*SERPINB7*	*CEBPE*
NT5C3A	*PPP1R1A*	*XKR9*	*ELFN1*
CRISP3	*PLP1*	*PARP12*	*RAB44*
PARM1	*PADI1*	*ISG20*	*IL12B*
LGALS3BP	*SERPINB11*	*SPC24*	*RAG2*
IL5RA	*SLC22A1*	*APOC1*	

IFIT5	*COL15A1*	*CD8B*
LPO	*CD1C*	*GLRB*
IFIT2	*RLBP1*	*MAP7D2*
GATA1	*THBS4*	*DNASE1L3*
IFIT3	*SLC6A5*	*MXD3*
DDX60	*NPPC*	*SPC25*
RNF213	*EGFLAM*	*AC104389.5*
HELZ2	*MMP27*	*ANKRD33B*
BPIFA1	*CHSY3*	*GRIA2*
TDO2	*NPPA*	*COL19A1*
CLCA1	*UGT1A6*	*RTP4*
RAG1	*CYTL1*	*TRIM71*
MMP8	*MYOZ2*	*LRR1*
DTX3L	*CSMD2*	*CDCA8*
EIF2AK2	*GABRD*	*PKMYT1*
PYHIN1	*UGT1A8*	*GPR83*
OAS1	*GPIHBP1*	*TMEM221*
MMP9	*TCP11*	*IL4I1*
ETV7		*LIPG*
CYP26A1		*CCNB3*
OAS2		*C5*
OAS3		*PLAC8*
IRF7		*CCL8*
TFF1		*GJA3*
UBA7		*PI3*
DUOX2		*TREX1*
OTOG		*OIP5*
CPXM1		*HOXC9*
TRIM6-TRIM34		
CAMP		
NTRK1		
STAT1		
TIMD4		
GPR31		
C1QTNF3-AMACR		
PARP9		
5S_RRNA_23163		
NLRP14		
F9		
HSH2D		
CMPK2		
SERPINC1		
HDC		
CD5L		
USP18		
OASL		
SAMHD1		
IFIH1		
CLEC1B		
DHX58		
AGR2		
TRIM22		

[at 5 dpi]

Common	Juvenile	Young Adult	Aged
LUM	*EPHA7*	*ZBP1*	*RARRES1*
MMP8	*MMP1*	*IL10*	*CA10*
MX1	*GPX6*	*CD70*	*CCL22*
TMEM255B	*TM4SF4*	*MACC1*	*CNTN6*
DTX3L	*SORCS1*	*PAX5*	*KCNIP3*
EIF2AK2	*LYZ*	*MYBPC1*	*AMBN*
RSAD2	*L1CAM*	*PLXDC1*	*ETV7*
WNT5B	*EMCN*	*ETNPPL*	*SFRP5*
MX2	*AZGP1*	*LGALS9C*	*CYP26A1*
PYHIN1	*COL3A1*	*DISP2*	*LRFN5*
KERA	*MFAP2*	*RBP7*	*MANSC4*
SHISA3	*SCN10A*	*DPM3*	*IFNG*
OAS1	*ELF5*	*TMIGD2*	*CD207*
MMP9	*LIX1*	*LGALS12*	*ATP13A5*
IFI44L	*COL5A3*	*SIGLEC1*	*MKX*
MYL7	*TFF2*	*CILP*	*PDCD1*
OAS3	*ACOT1*	*FXYD2*	*CD69*
FAP	*DSG3*	*LY6E*	*LY6H*
MYL4	*TFF1*	*MELTF*	*VIP*
TFF3	*MS4A2*	*ATP13A4*	*KANK4*
ARPP21	*SNAP25*	*THRSP*	*AC090227.1*
SCG2	*ST6GALNAC1*	*COX20*	*LNP1*
MXRA5	*TNFAIP6*	*ACOT6*	*CRYBG2*
KCNK2	*NRK*	*S100A9*	*ANKRD35*
SCG3	*HEPACAM2*	*S100A8*	*CD80*
CLEC4G	*TRANK1*	*VIT*	*NOXO1*
C17ORF78	*KRT23*	*RAMP3*	*C4BPB*
CD177	*MPO*	*RBPJL*	*HTR2B*
TELOMERASE-VERT	*CXCL14*	*NYAP2*	*THY1*
RETNLB	*SNX31*	*DGKB*	*UBE2L6*
UBA7	*EPX*	*HAAO*	*C4A*
AC140504.1	*ADCY8*	*UCP2*	*SLC5A5*
IFI6	*ADAMTS12*	*JCHAIN*	*CLGN*
SAMD9L	*HAPLN3*	*OC90*	*SLC22A13*
HERC6	*ADAMTS17*	*SLC7A4*	*PROKR1*
HERC5	*CAPN9*	*NEURL1B*	*THPO*
CA4	*MDK*	*DUOX2*	*FLRT1*
FAM196B	*TNN*	*MAP1LC3C*	*C10ORF10*
TRIM6-TRIM34	*HAS2*	*CA6*	*GBP1*
PCK1	*CD300LB*	*MALRD1*	*NXPH2*
EPSTI1	*SLC26A10*	*SPP1*	*TINAG*
CAMP	*FCER1A*	*S100A12*	*SLC25A48*
GPR158	*CPXM1*	*PLK5*	*CTBS*
APOBEC3G	*OTX2*	*BATF3*	*CCDC30*
DDX58	*GPC5*	*P2RY12*	*GPR39*
TIMD4	*CPA3*	*CDKN2A*	*RIC3*
ISG15	*ST18*	*LRRC75B*	*IL33*
PARP15	*CUBN*	*PVRIG*	*LGI1*
IFI44	*KCNK13*	*GZMA*	*CGREF1*
PARP9	*LVRN*	*EPHX4*	*TMPRSS11A*
PARP14	*PLA2G4D*	*XKR9*	*SCD5*
BPIFB1	*DDX11*	*CHIT1*	*PPBP*
PARP12	*ANXA10*	*SULT2B1*	*ADGRF1*
NLRP14	*SHCBP1*	*DHRS9*	*TCN1*

NA	*RFLNB*	*APOC1*	*STAC2*
NT5C3A	*GJB1*	*COL8A1*	*ACSBG1*
GAP43	*F9*	*PADI4*	*CDH16*
HSH2D	*RIT2*	*DNASE1L3*	*AC104389.5*
KLHDC8A	*KIT*	*MATN4*	*IL7R*
CMPK2	*GLRB*	*SLC22A1*	*GRIA2*
PCOLCE2	*CD226*	*CYGB*	*GSG1L2*
LTF	*PLCB1*	*CYSRT1*	*COL19A1*
ZNF215	*PADI1*	*RIMS3*	*C1R*
FGF13	*SERPINB11*	*RAB42*	*SERPINC1*
SPC25	*LY6L*	*PLAC1*	*DOC2A*
CRISP3	*CFI*	*PWWP2B*	*C1S*
CCL13	*PCDH12*	*TREX1*	*SEMA3E*
RTP4	*COL15A1*	*MUC15*	*PLA2G7*
LGALS3BP	*COL11A2*	*GYG2*	*LYPD6*
IFIT5	*HDC*	*NKAIN1*	*GPR83*
ABCA13	*IL5RA*		*SAMHD1*
IFIT2	*LPO*		*NDST3*
USP18	*C1ORF54*		*CLEC1B*
OASL	*CD1C*		*PLAC8*
IFIT3	*PCDH17*		*RGS1*
DDX60	*RNF213*		*KCNMB2*
IFIH1	*ADAMTS6*		*PTCHD1*
BPIFA1	*THBD*		*AP1S3*
DHX58	*EDNRB*		*ENPP3*
PI3	*CHSY3*		*ANKRD55*
PGLYRP1	*C1QTNF6*		*OLR1*
TRIM22	*TESPA1*		
	PDGFD		
	UGT1A6		
	SLITRK6		
	CLCA1		
	NOS2		
	TRPM6		
	EPHB2		
	AHSP		
	GABRD		
